# Supplementary material for: Novel Driver Strength Index highlights important cancer genes in TCGA PanCanAtlas patients
Source: PeerJ. 2022 Aug 11;10:e13860. doi: 10.7717/peerj.13860 (PMC9375969; doi:10.7717/peerj.13860)
Supplement: Supplemental Information 2 [file peerj-10-13860-s002.zip › COHORTS/genes plots/detailed/2021_8_16_14_9_distribution_events_detailed_females_40.pdf]

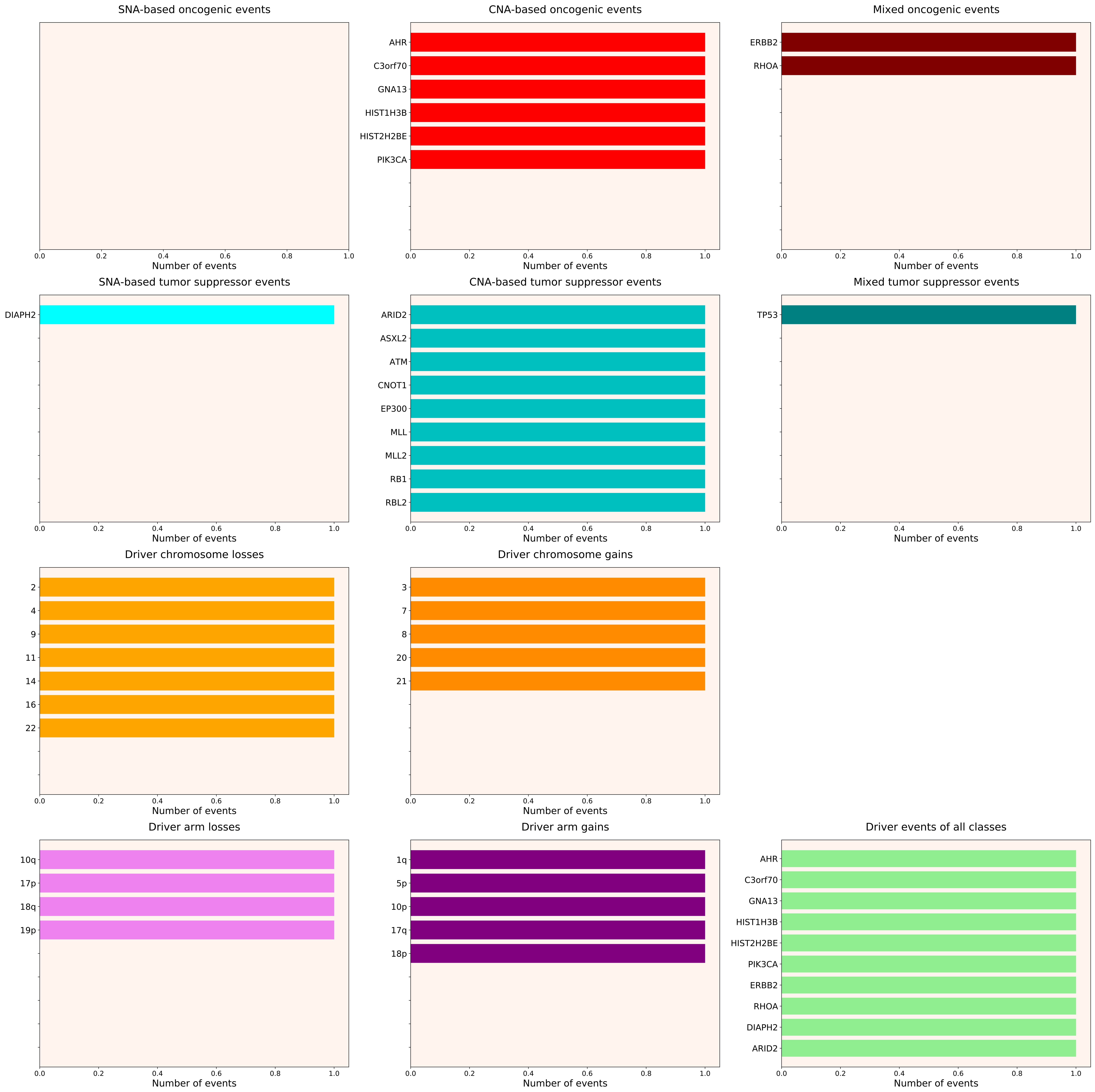
SNA-based oncogenic events
0.0
0.2
0.4
0.6
0.8
1.0
Number of events
CNA-based oncogenic events
AHR
C3orf70
GNA13
HIST1H3B
HIST2H2BE
PIK3CA
0.0
0.2
0.4
0.6
0.8
1.0
Number of events
Mixed oncogenic events
ERBB2
RHOA
0.0
0.2
0.4
0.6
0.8
1.0
Number of events
SNA-based tumor suppressor events
DIAPH2
0.0
0.2
0.4
0.6
0.8
1.0
Number of events
CNA-based tumor suppressor events
ARID2
ASXL2
ATM
CNOT1
EP300
MLL
MLL2
RB1
RBL2
0.0
0.2
0.4
0.6
0.8
1.0
Number of events
Mixed tumor suppressor events
TP53
0.0
0.2
0.4
0.6
0.8
1.0
Number of events
Driver chromosome losses
2
4
9
11
14
16
22
0.0
0.2
0.4
0.6
0.8
1.0
Number of events
Driver chromosome gains
3
7
8
20
21
0.0
0.2
0.4
0.6
0.8
1.0
Number of events
Driver arm losses
10q
17p
18q
19p
0.0
0.2
0.4
0.6
0.8
1.0
Number of events
Driver arm gains
1q
5p
10p
17q
18p
0.0
0.2
0.4
0.6
0.8
1.0
Number of events
Driver events of all classes
AHR
C3orf70
GNA13
HIST1H3B
HIST2H2BE
PIK3CA
ERBB2
RHOA
DIAPH2
ARID2
0.0
0.2
0.4
0.6
0.8
1.0
Number of events
